# Land use types influenced avian assemblage structure in a forest–agriculture landscape in Ghana

**DOI:** 10.1002/ece3.3355

**Published:** 2017-09-18

**Authors:** Justus Precious Deikumah, Richard Kwafo, Vida Asieduwaa Konadu

**Affiliations:** ^1^ Department of Conservation Biology and Entomology School of Biological Sciences, University of Cape Coast Cape Coast Ghana

**Keywords:** agricultural land type, avian assemblage, forest specialists, forest‐agriculture landscape, functional groups, native trees

## Abstract

The conservation of biodiversity within tropical forest regions does not lie only in the maintenance of natural forest areas, but on conservation strategies directed toward agricultural land types within which they are embedded. This study investigated variations in bird assemblages of different functional groups of forest‐dependent birds in three agricultural land types, relative to distance from the interior of 34 tropical forest patches of varying sizes. Point counts were used to sample birds at each study site visited. Data from counts were used to estimate species richness, species evenness, and Simpson's diversity of birds. Mean species richness, evenness, and diversity were modeled as responses and as a function of agricultural land type, distance from the forest interior and three site‐scale vegetation covariates (density of large trees, fruiting trees, and patch size) using generalized linear mixed‐effect models. Mean observed species richness of birds varied significantly within habitat types. Mean observed species richness was highest in forest interior sites while sites located in farm centers recorded the lowest mean species richness. Species richness of forest specialists was strongly influenced by the type of agricultural land use. Fallow lands, density of large trees, and patch size strongly positively influenced forest specialists. Insectivorous and frugivorous birds were more species‐rich in fallow lands while monoculture plantations favored nectarivorous birds. Our results suggest that poor agricultural practices can lead to population declines of forest‐dependent birds particularly specialist species. Conservation actions should include proper land use management that ensures heterogeneity through retention of native tree species on farms in tropical forest‐agriculture landscapes.

## 1 INTRODUCTION

The significance of matrix or land type near wildlife habitats has been recognized globally (Deikumah, McAlpine, & Maron, 2014; Kennedy, Marra, Fagan, & Neel, 2010; Ruiz‐Guerra, Renton, & Dirzo, 2012). A matrix can be a major source of feeding and breeding site for wildlife (Antongiovanni & Metzger, 2005); a link permitting movement of wildlife between habitats (Devictor & Jiguet, 2007) and a temporary or permanent habitat for some species (Cline & Hunter, 2016). Therefore, understanding how changes in a matrix impacts biodiversity is necessary to develop conservation strategies.

The persistence of wild animals occupying a forest patch can be influenced by the type of matrix that surrounds that patch (Dunford & Freemark, 2005). Land use type/matrices such as roads (Holden, 2015; Marcantonio, Rocchini, Geri, Bacaro, & Amici, 2013), commercial and subsistence agricultures (Bolwig, Pomeroy, Tushabe, & Mushabe, 2006; Sodhi et al., 2010), mining areas (Macdonald et al., 2015; Tapia‐Armijos, Homeier, Espinosa, Leuschner, & de la Cruz, 2015), infrastructure expansion, and urban development (Delphin, Escobedo, Abd‐Elrahman, & Cropper, 2016; Villaseñor, Driscoll, Escobar, Gibbons, & Lindenmayer, 2014) can influence wildlife populations in nearby habitats. Such land use types often differ in the pressures they exert on wild animals (Lira, Tambosi, Ewers, & Metzger, 2012). Recent studies in southwest Ghana suggested that mining matrices adjacent forest remnants negatively influenced the abundance of forest‐dependent birds and disrupted the functional composition of bird communities (Deikumah, McAlpine, & Maron, 2013). Similarly, land use for agricultural purposes such as shade–grown cocoa and coffee was reported to harbor high biodiversity due to the presence of diverse high canopy forming species, complex forest structure, and absence of invasive exotic weeds according to (Siebert, 2002). Such land types often provide suitable habitats for native fauna and were especially good for birds. In contrast, sun‐grown monocultures (e.g., cocoa, coffee, and oil palm) were found to have adverse effects on biodiversity due to homogenization and presence of invasive weed species (Philpott et al., 2008). In most tropical forest areas, agricultural lands are predominant types of land use around native forest patches (Gonthier et al., 2014; Harvey & Villalobos, 2007; Perfecto & Vandermeer, 2008), but their influence alone on the persistence of faunal diversity in some tropical regions is poorly understood.

Agricultural lands can be essential components in biodiversity conservation within tropical forest–agricultural landscapes if properly managed (Rodrigues et al., 2013). The isolation of protected areas as the sole means of protecting biodiversity is insufficient given the current trend in land use dynamics (Siebert, 2002). Studies demonstrated the potentials of diverse agricultural areas in supporting biological diversity and stresses their integration in conservation strategies (Perfecto & Vandermeer, 2008; Schroth, 2004). Agricultural lands when properly managed will not only support a large number of biodiversity but also serve as safe corridors that will permit dispersal of wildlife between patches (Norris, 2008). Perfecto and Vandermeer (2002) proposed that managed agricultural areas were equally important as the forest patches they surround. In their study, they found that species richness of ground‐foraging ants in a well shaded organic cocoa farm did not differ from that of a nearby montane forest. Similarly, Harvey et al. (2008) confirmed the conservation value of agricultural lands, mainly areas that retained an abundant native tree cover. Such areas as suggested, often exhibited structural heterogeneity while providing habitat and resources for native fauna species (Fahrig et al., 2015).

Over the past decades, many native forest areas have come under intense pressure due to anthropogenic activities and invasions (Munro, Fischer, Wood, & Lindenmayer, 2009). Clearing for agriculture and poor agricultural practices with associated pressures have had severe consequences on biodiversity and have led to the loss of many native wildlife species globally (Posa & Sodhi, 2006). Over 80% of forest loss globally has been linked to agriculture (FAO 2010). In Latin America, commercial agriculture accounted for two‐thirds of forest loss, while in Africa and Asia commercial and subsistence agriculture were the primary drivers for more than one‐third of forest loss (FAO 2010). In West Africa, the conversion of forest habitats to farms coupled with poor agricultural practices has exacerbated the trend in forest biodiversity loss in these regions (Gibbs et al., 2010; Gockowski & Sonwa, 2011; Waltert, Bobo, Sainge, Fermon, & Mühlenberg, 2005). These pose major concerns for the conservation of tropical biodiversity within forest–agricultural landscapes.

Several studies relating birds to their environments in forest–agricultural landscapes globally suggest that variations in agricultural land use can have different impacts on forest‐dependent bird assemblages (Carrara et al., 2015; García & Martínez, 2012). However, only a few of such studies were conducted in tropical Sub‐Saharan Africa (Phalan, 2010; Sekercioglu, 2002; Söderström, Kiema, & Reid, 2003). In West Africa, the effects of agricultural land use on tropical forest fauna are poorly understood (Beier, Van Drielen, & Kankam, 2002). Little is known about how forest‐dependent birds are affected by traditional agricultural land types within which forest habitats are embedded in the biodiversity hot spot Upper Guinea Forest Zone. It remains unclear how avifaunal assemblages fair in tropical forest–agricultural landscapes with changing agricultural practices and management regimes. In this study, we investigated the effects of agricultural land types on bird assemblage structure in 34 Upper Guinean forest remnants of varying sizes in Ghana. We compared bird assemblages in different farm types and fallow lands relative to adjacent forest remnants and with distance from farm center toward the interior of each forest patch. We predicted that adjacent agricultural land types with vegetation structure similar to the forest patch would positively influence avian assemblage structure and that bird assemblages will change along changing vegetation gradients from farm toward forest interior. We also predicted that species richness of forest‐dependent birds especially forest specialists and food specialists (insectivores and frugivores) would be negatively impacted by land use modification that causes a patch‐matrix contrast in forest and agricultural land type.

## 2 METHODS

### 2.1 Study area

**Figure 1:**
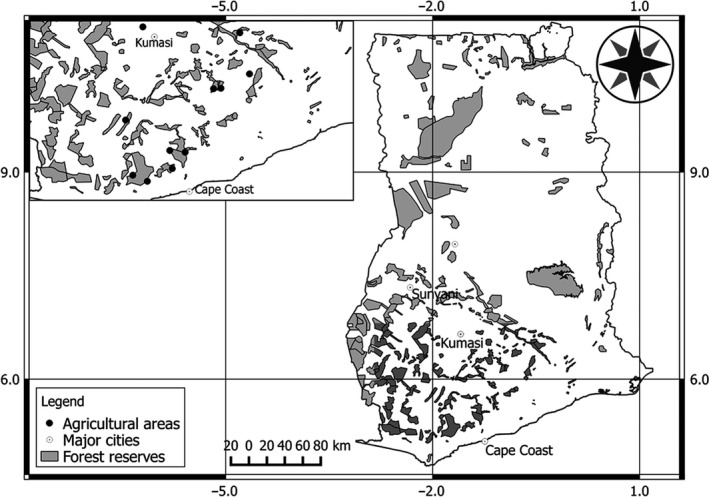
Map showing bird survey sites located within forest–agricultural landscape in southwest Ghana

The study was conducted in the Upper Guinean forest in West of Ghana (Figure 1). Bordering the Gulf of Guinea, Ghana lies within the coordinates 3°5′W‐1°10′E; 4°35′N‐11°N, and covers an area of 238,535 km^2^. Vegetation is characterized by an evergreen and semi‐deciduous forests consisting of trees such as mahogany, silk cotton tree, and ebony. The northern part of the country is covered by savannah grassland with scattered trees. The major rainy season begins from March extending to July but peaks in June while the minor season lasts from September to November.

Ghana falls within four biogeographic zones: Sudan in the north, Guinea‐Congolian in the southwest, Guinea‐Congolian/Sudanian transition zone in the center and southeast, and Volta in the east (Hawthorne & Abu‐Juam, 1995). The forest areas of southwest Ghana are highly fragmented due to illegal logging activities, forest clearing for agricultures, and rapid population growth around forest areas (Holbech, 2009). Surrounding the forest fragments is a sea of varying land use dominated by small farms, fallow lands, and commercial monoculture plantations (teak, rubber). The predominant type of agricultural land use is the cultivation of cocoa, oil palm, teak, cassava, plantain, maize, banana, rice, yam, and vegetables. Cocoa farms usually have native trees retained on them as such have structures that are similar to native forest areas. In this study, we selected three agricultural land types representative of the wider farm types in the Upper Guinean forest areas.

### 2.2 Experimental design

A total of thirty‐four (34) forest patches of varying sizes ranging from 2.3 to 588 km^2^ were selected in this study. Forest patches used in this study were divided into three categories; small (≤3.8 km^2^), medium (4.2–8.0 km^2^), and large sized (≥15.6 km^2^) forest patches. We selected large (*N* = 10), medium (*N* = 13), and small (*N* = 11) sized forest patches. Considerations of patch size in the study design were based on conclusions from the equilibrium theory of island biogeography (MacArthur & Wilson, 1967). Though this theory played remarkable roles in the design of reserves to conserve species, several authors have criticized the relevance of large reserves on theoretical and empirical viewpoints (Margules, Higgs, & Rafe, 1982; Simberloff & Abele, 1976). Controversies are centered around the importance of small reserves, particularly the capacity of two or more reserves to support biodiversity when their combined area is equal to that of a single large reserve. In our case, we also examine, the importance of patch size asking whether or not the size of a forest patch matters for forest birds in a forest–agricultural landscape.

Each forest patch category had at least two different agricultural land types surrounding it. In the agricultural lands, one site was located and categorized as “farm center.” At 300 m from the farm center toward the forest patch, another site was located and categorized as forest “edge site.” Forest edge sites were at 50 m wide. A third site was located closer to the interior (at least 500 m from the forest boundary) and categorized as forest “interior site.” Edge and interior sites were carefully distributed in and around each forest patch. Agricultural land types selected in this study are categorized as cocoa farms with large trees retained on them, monoculture plantations (teak and rubber plants), and fallows (abandoned farmlands). Agricultural land types used in this study were distributed around the different sized forest patches. We sampled cocoa farms (*N* = 12), monoculture plantations (*N* = 12), and fallows (*N* = 10). The size of agricultural lands was ≥1.5 km^2^.

### 2.3 Bird surveys

Bird surveys were conducted between December 2014 and March 2015 and further sampling between October 2016 and March 2017. Point count method was adopted in this study. At each site, three sampling locations were randomly chosen, and all birds within a 50 m radius from a single observer within a 10‐min sampling period were identified and recorded. Sampling locations at each site were at least ≥200 m apart. A 5‐min rest period was allowed for birds to begin normal behavior before census started due to the initial disturbance caused by the observer. Sampling locations were visited twice throughout the whole survey period. Counts were made twice a day in each location between 05:00 and 09:00 hrs and late afternoon from 14:00 to 17:00 hrs. Flyovers were not considered for analysis but were noted. All observations beyond 50 m were discarded from the final analysis. Efforts were made to avoid double counting of birds that could move between count stations. Bird calls that were unfamiliar were taped in the field to confirm identification later with experts. All bird surveys were conducted by the same observer (RK). At each study site, the total number of individual species detected was used to create a species abundance database. Observed species richness was calculated by pooling all visits from each study location together. All counts were conducted during periods without heavy rains or strong winds.

### 2.4 Vegetation surveys

Local vegetation surveys at each site were conducted to characterize the composition and structure of vegetation in the study area. Vegetation characteristics were quantified using protocols from literature (Naidoo, 2004; Rodewald, 2003). Vegetation characteristics estimated in this study were density of large trees, percentage canopy cover, percentage shrub cover, percentage ground cover, number of flowering, and fruiting trees (see Table 1 for description). A 20 m × 20 m quadrat was randomly placed at each bird survey location per study site. Within this quadrat, all trees in the size range of ≥30–60 cm diameter at breast height (DBH) were counted, measured, and categorized as large trees. The estimate was used in computing for density of large trees as the number of trees per hectare. Within the same quadrat, all flowering and fruiting trees were identified and counted. Percentage canopy, ground, and shrub cover were estimated by visual inspection and computed as the average number of three estimates to represent percentages of canopy, ground, and shrub cover. Canopy and shrub cover estimates were not used in the final analysis because they correlated. Ground cover was excluded from final analysis because it had no significant influence on any of the response variables.

**Table 1:** Description of landscape and site‐scale variables

Variables	Definition	Unit	Description
Landscape variable			
Farm type	Agroforestry	—	Approximately ≥5‐year‐old cocoa plantations with large shade trees of 1–4 km^2^
			Monoculture plantations up to 5 years
	Fallow		Abandoned farmlands >5 years with large trees retained on them
Site‐scale variable			
Vegetation variable	Percentage shrub cover	Percent	Understory foliage projected cover of small plants and young trees
	Density of large trees	Number/ha	Number of trees with DBH ≥30–60 cm per hectare (ha)
	Percentage canopy cover	Percent	% of fixed area covered by crowns of each tree when observed from above
	Percentage ground cover	Percent	Lower level plants, litter, bare ground
	Flowering trees	Count	Total of all flowering plants (shrubs, trees)
	Fruiting trees	Count	Total of all fruiting plants (shrubs, trees)
	Patch size	km^2^	Size of individual forest patches

### 2.5 Ecological traits

Ecological traits of all birds recorded were gathered from literature (Bennun, Dranzoa, & Pomeroy, 1996; Borrow & Demey, 2010). Birds were grouped according to their habitat and food preferences, respectively. Four habitat preference categories were identified as follows: forest generalists, forest specialists, forest visitors, and open habitat species (see Table 2 for description). Birds were further categorized into the following four foraging preference categories: granivores, frugivores, insectivores, and nectarivores based on diet information obtained from literature (Deikumah et al., 2014; Holbech, 2009; see Table S1 for description).

**Table 2:** Categories of bird used in this study (Bennun et al., 1996; Borrow & Demey, 2010)

Species category	Description
Forest generalists	Species can be found in undisturbed forest but are also regularly found in forest edges
Forest specialists	Characteristic of the interior of undisturbed forest
Forest visitors	Species that are often found in forest but are not dependent upon it
Open habitat species	Normally breed outside forest

### 2.6 Data analysis

Species richness, species diversity, and species evenness were computed for all 34 study site. Species richness was estimated using EstimateS version 8.2.0. Abundance‐based species richness estimators Chao1 was used to compute estimated species richness using a bias‐corrected formula (Colwell, 2005). Chao1 is a nonparametric species estimator used for estimating the true total number of species in a given area based on multiple samples and is a practically useful estimator of species richness when there are undetectable species in a very diverse assemblage (Colwell & Coddington, 1994). Species richness within bird functional groups was calculated. Species richness, evenness, and diversity were compared in a two‐factor analysis of variance (ANOVA) for all birds recorded in agricultural areas, forest edge, and interior sites. Species richness of bird functional groups was also compared among the different study sites. Before analysis begun, data were screened for normality (Shapiro–Wilks test) and equality of variance (Bartlett test). Significance was set at α = .05. Data were log‐transformed where necessary before used in the final analysis.

Included in the final analysis were twelve response variables: observed species richness (sob), estimated species richness (Chao1), species evenness, Simpson's diversity, observed species richness for foraging preference groups (insectivores, frugivores, granivores, and nectarivores), and observed species richness for habitat preference groups (forest specialists, forest generalists, forest visitors, and open habitat species). Using Spearman's correlation coefficient matrix, all explanatory variables were tested for collinearity. Paired explanatory variables that had a greater possibility of influencing variations in bird assemblages were used for further analysis (see Table S2).

All twelve responses were modeled as a function of five explanatory variables using generalized linear mixed‐effect models. Mixed‐effect models were performed using the “lme4” package (Pinheiro, Bates, DebRoy, & Sarkar, 2012) in a model averaging framework with “MuMln package” in R version 3.1.1 (Development CoreTeam R 2013). Mixed‐effect models offer a more robust approach to quantify the relationship between response and explanatory variables and therefore were ideal for analyzing hierarchically structured and nested data like ours (Baayen, Davidson, & Bates, 2008).

We generated 64 models with all possible combinations of predictors and responses. Model uncertainty was high between 4 and 15 models in a 95% confidence set (summed Akaike weights (Σω_*i*_) = 0.95) for the response variables. The model of best fit was selected from the full models based on the Akaike Information Criterion values (Full model = response ~ distance*land use + large trees + fruiting trees + patch size + (1|site)) (Burnham, Anderson, & Huyvaert, 2011). Included in the models was an interaction term between distance to interior and land use type, where they were added as main effects. The variable “site,” was included in the model as a random effect. Across all models, the model‐averaged parameter estimates and standard errors for the estimates for each of the response variables were calculated in a confidence set (Burnham & Anderson, 2002). All explanatory variables were ranked according to their importance in influencing each of the response variables. This was performed using the summed Akaike weights (Σω_*i*_) from all model combinations where the variable of concern occurred. The higher the Σω_*i*_ value, the more important the variable compared to other variables (Burnham & Anderson, 2002).

To ensure that study sites were spatially independent, we accounted for spatial autocorrelation (Dale & Fortin, 2002). To test for spatial autocorrelation, a spline correlogram of the model residuals of full models for all response variables was constructed. The spline.correlog function in the ncf package was used to produce the spline correlogram with 10,000 permutations (Bjørnstad, 2008) based on the Morans I index (see Fig. S1).

## 3 RESULTS

### 3.1 Species richness

**Figure 2:**
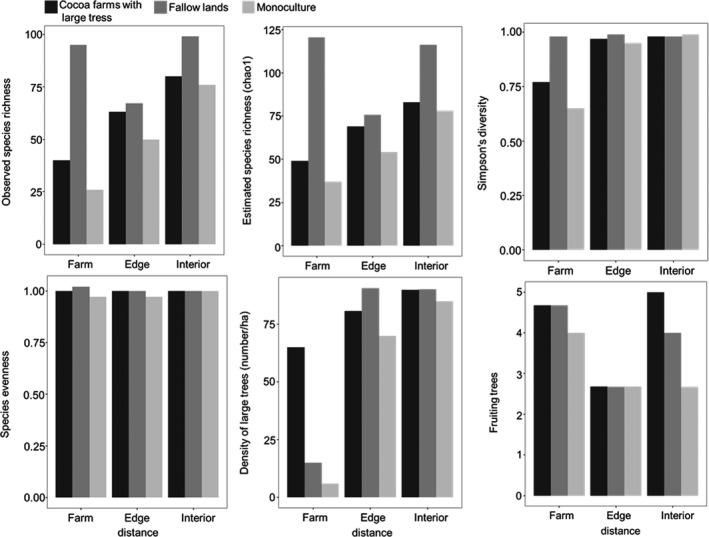
Plots of diversity indices and vegetation covariates measured across all forest and agricultural land types

A total of 154 species of birds belonging to 35 families and 82 genera were recorded in the surveys. Observed and estimated species richness between study locations did not differ and showed similarity in patterns of change and response to environmental variables. Therefore by convenience, we select observed species richness on which we present results and base our discussions and conclusions. Three red list species of conservation concern were recorded as follows: hooded vulture (*Necrosyrtes monachus*)*,* green‐tailed bristlebill (*Bleda eximia*), and rufous‐winged illadopsis (*Illadopsis rufescens*; Birdlife International 2017). Mean observed species richness varied significantly for the different agricultural land use (*F*_2,135_ = 5.18, *p* < .05), and with distance from farm center toward the forest interior (*F*_2,135_ = 5.55, *p* < .05). Mean observed species richness was highest in fallow lands followed by cocoa farms with large trees and lowest in monoculture plantations (Figure 2). Mean observed species richness was highest in forest interior sites while sites located in farm centers recorded the lowest mean species richness (Figure 2).

Significant differences were observed in mean richness among avian functional groups with respect to agricultural land use (*F*_2,135_ = 10.37, *p* < .05). Species richness of forest specialists was highest in fallow lands and lowest in monoculture plantations.

Species evenness varied significantly within the different agricultural land types (*F*_2,135_ =* *5.69, *p* < .05) as well as with distance from farm toward forest interior (*F*_2,135_ = 19.65, *p* < .05; Figure 2).

Species diversity varied regarding land use (*F*_2,135_ = 4.06, *p* < .05) and with distance from farm center toward forest interior (*F*_2,135_ = 27.46, *p* < .05). Species diversity was highest in forest interior compared to forest edges and lowest in farm centers (*p* < .05; Figure 2).

### 3.2 Variations in vegetation characteristics within agricultural land use

All three site‐scale vegetation covariates used in the final analysis varied in the different agricultural types and with distance toward forest interior (Figure 2). The density of large trees was highest in sites located in the forest interior compared to forest edges but lowest in farms (*F*_2,135_ = 179.10, *p* < .05; Figure 2). Forest edges near fallow lands recorded the highest number of large trees while there were fewer large trees in monoculture plantations (Figure 2). Mean number of fruiting trees varied among study sites but was low (Figure 2).

### 3.3 Model averaging and effects of landscape and site‐scale variable on responses

**Figure 3:**
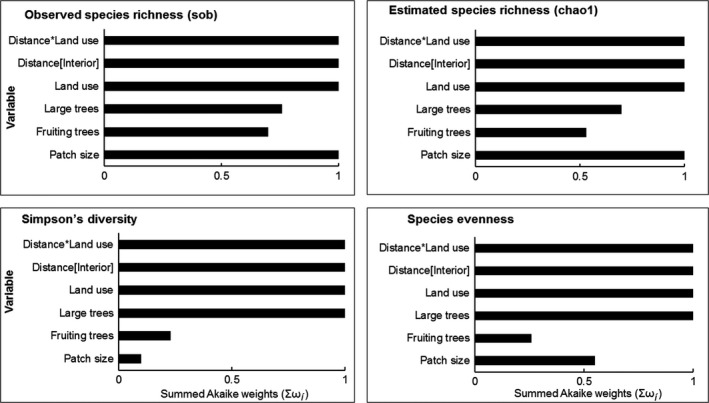
Relative importance of four environmental variables on diversity indices (observed species richness (sob), estimated species richness (chao1), Simpson's diversity index, and species evenness)

**Figure 4:**
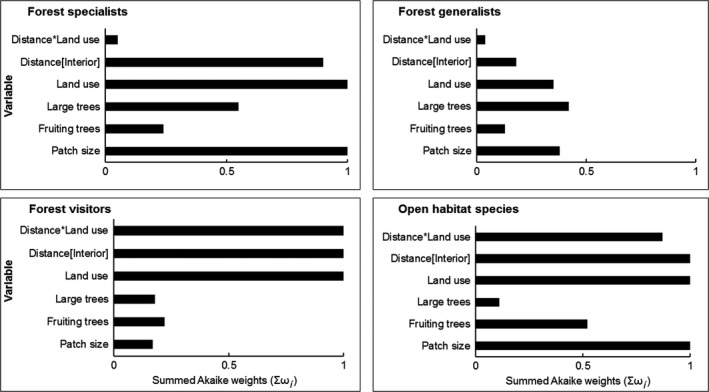
Relative importance of four environmental variables on habitat preference (forest specialists, forest generalists, forest visitors, and open habitat species)

**Figure 5:**
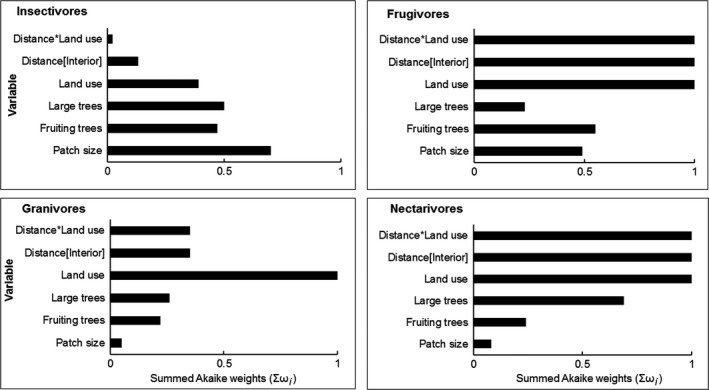
Relative importance of four environmental variables on foraging preference (insectivores, frugivores, granivores, and nectarivores)

Twelve response variables were modeled as a function of five explanatory variables at 95% confidence level (i.e., summed Akaike weight; Σω_*i*_ = 0.95). Size of the forest path, distance, land use, and distance‐land use interaction was important variables that influenced mean observed richness (Σω_*i*_ = 1.0; Figure 3). These variables along with large trees were important in influencing species evenness and diversity (Figure 3). Fallow lands and size of forest patch ranked highest in influencing richness of forest specialists (Σω_*i*_ = 1.00; Figure 4). Distance toward the forest interior ranked high with AIC weight (Σω_*i*_ = 0.90). Distance [toward the farm] and land use [monoculture plantation] were important predictors that influenced richness of forest visitors with (Σω_*i*_ = 1.00), respectively (Figure 4). Density of large was important in influencing forest specialists but with low summed AICc weight (Σω_*i*_ = 0.57; Figure 4). Fallow lands and density of large trees positively influenced forest specialists (Table 3). Fallow lands were important in influencing the richness of insectivorous bird. Distance toward forest interior and the type of land use ranked highest in influencing frugivorous birds (Σω_*i*_ = 1.0; Figure 5).

**Table 3:** Model‐averaged coefficient estimates (±*SE*) across the 95% confidence interval of models for all explanatory variables

Responses	Explanatory variables					
	Distance*land use	Distance[Interior]	Land use	Large tree	Fruiting trees	Patch size
*Diversity indices*						
Observed species richness	**0.54 ± 0.15**	0.07 ± 0.10	0.13 ± 0.17	0.00 ± 0.00	0.03 ± 0.03	**−0.49 ± 0.16**
Estimated species richness (chao1)	**0.51 ± 0.15**	**0.07 ± 0.11**	0.26 ± 0.18	**0.01 ± 0.00**	0.10 ± 0.02	−0.52 ± 0.15
Simpson's diversity	**0.08 ± 0.03**	**0.04 ± 0.02**	0.01 ± 0.03	**−0.01 ± 0.00**	0.02 ± 0.02	−0.01 ± 0.01
Species evenness	**0.09 ± 0.03**	−0.04 ± 0.02	−0.01 ± 0.03	0.01 ± 0.00	0.00 ± 0.00	−0.02 ± 0.02
*Forest habitat preference*						
Forest specialists	−0.07 ± 0.04	**0.14 ± 0.07**	**0.12 ± 0.06**	0.01 ± 0.00	0.00 ± 0.00	−0.33 ± 0.08
Forest generalists	0.01 ± 0.05	0.00 ± 0.02	−0.08 ± 0.14	0.01 ± 0.01	0.00 ± 0.00	−0.11 ± 0.17
Forest visitors	**−0.28 ± 0.12**	0.04 ± 0.08	**0.31 ± 0.15**	0.00 ± 0.00	−0.04 ± 0.01	−0.03 ± 0.09
Open habitat species	−0.40 ± 0.25	−0.12 ± 0.13	0.13 ± 0.16	0.00 ± 0.00	−0.02 ± 0.03	**−0.50 ± 0.30**
*Foraging preference*						
Insectivores	0.01 ± 0.05	0.00 ± 0.05	0.06 ± 0.09	−0.00 ± 0.00	−0.01 ± 0.02	−0.07 ± 0.10
Frugivores	**−0.35 ± 0.10**	**0.20 ± 0.07**	**0.45 ± 0.12**	−0.00 ± 0.00	**−0.01 ± 0.01**	−0.08 ± 0.11
Granivores	0.10 ± 0.17	−0.04 ± 0.09	0.31 ± 0.21	0.00 ± 0.00	0.00 ± 0.00	−0.01 ± 0.05
Nectarivores	**0.03 ± 0.19**	**0.50 ± 0.17**	**1.04 ± 0.19**	0.00 ± 0.00	−0.02 ± 0.01	0.00 ± 0.04

Values in bold characters indicate a significant coefficient.

Results from spline correlogram of Moran's similarity index for model residuals suggested no significant spatial autocorrelation among sites except for models for forest specialists’ species and granivores where there was weak positive spatial autocorrelation (Fig. S1).

## 4 DISCUSSION

This study revealed that the type of agricultural land use surrounding a tropical rainforest patch could significantly influence bird assemblage structure in forest–agricultural landscapes. The study found that species richness, evenness, and bird diversity were high in forested areas. Different avian functional groups in forest patches were strongly influenced by the type of agricultural land use, patch size, and distance gradient toward patch interior. The study also found a significant influence of large trees either in the forest or those retained on farmlands on avifauna assemblages. This study suggests that to support forest bird diversity in forest–agricultural landscapes, proper agricultural practices that include maintenance of large native trees on farms is crucial. However, this should take into account habitat requirements for the different functional groups.

### 4.1 Responses to land use and distance from forest interior

All three diversity indices were high in forest areas compared to agricultural lands. The importance of forests for maintaining and conserving biodiversity is well known (Loo Judy, 2009; Thiollay, 1995). High species diversity in tropical forest areas has been linked to complex vegetation structure and composition that support the needs of vast organisms that depend on it (Connell, 1978). When forest areas are rich in resources to support life, more species thrive. Many birds rely on forest areas for survival, especially for species that are closely associated with tropical forest and depend solely on them, they will disappear if all forest areas are lost (Sodhi, Liow, & Bazzaz, 2004). The Upper Guinean forest patch widely known for its rich rainforest plant diversity, closed canopies and diverse shrub understory, provides suitable habitats for many species especially birds. Structural and taxonomical diversity in such habitats provide greater opportunities for resource allocation and therefore supports greater richness and diversity of birds (Nadkarni, 1994). High species richness and diversity of birds recorded in these forest areas are not surprising.

We found that species richness and diversity of forest specialists were significantly lower in areas where monoculture plantations were located adjacent to the forest patches than areas where cocoa farms with large trees and fallow lands were located. When natural areas are replaced with monocultures, there is a complete change in vegetation structure accompanied by the loss of structural complexity. The uniformity in vegetation pattern reduces diversity while affecting bird community composition negatively. In many cases, generalists and habitat edge species are unaffected while specialists are heavily impacted with these modifications. Forest specialists are highly sensitive to forest conversion and land use change; therefore, they exhibit high habitat specificity and dependence on interior habitats (Carrara et al., 2015; Korpela, Hyvönen, & Kuussaari, 2014). This finding provides evidence that monoculture plantations surrounding forest patches can negatively affect forest‐dependent birds particularly forest specialists. This is consistent with earlier studies that found forest specialists birds to have shown greater sensitivity to varying agricultural land use, particularly monocultures, and that a slight change in vegetation characteristics could impact bird assemblages (Maas et al., 2009; Schulze & Riedl, 2008). In many tropical regions, forest‐dependent birds are the most threatened due to their sensitivity to human disturbance through unfriendly agricultural practices (Naidoo, 2004).

This current study also revealed that the adjacent land use around a forest patch was important for two avian foraging guilds (i.e., insectivores and frugivores); three habitat preference groups namely forest specialists, forest visitors, and open habitat species. All two foraging preference groups were more species‐rich in forest areas adjacent to cocoa farms with large trees and fallow lands compared to monoculture plantations. This is probably because farms, where trees are retained, appear structurally, and floristically similar to the adjacent forest patch as observed in this study. Such areas may have potential nesting, perching, and foraging sites for birds (Sekercioglu, Loarie, Brenes, Ehrlich, & Daily, 2007). This study corroborates earlier studies globally, for example Geist and Lambin (2002), McLaughlin (2011). For instance, Caprio, Ellena, and Rolando (2009) assessed predictors of bird diversity in a deciduous forest in Italy and reported that density of oak significantly influenced specialist species. They suggested that retaining native oak trees in a deciduous woodland originally dominated by oak was a key predictor for maintaining the diversity of specialist species.

We found more forest visitors in areas adjacent monoculture plantations compared to other agricultural land types considered in this study. Although forest visitors do not necessarily require forests for survival, they may utilize the forest as corridors (Farwig, Böhning‐Gaese, & Bleher, 2006). Their presence in the forest interior probably signified reduced biotic integrity of forests resulting from unsustainable agricultural land use and practices (Lindenmayer, Cunningham, Donnelly, Nix, & Lindenmayer, 2002).

### 4.2 Effects of vegetation covariates on birds

There was a strong association between species richness of birds and patch size. The size of our sampled forest patches was important for forest‐dependent species especially forest specialists. Larger forest patches in this study supported larger numbers of forest‐dependent species than did smaller sized patches. This result may have been probable because, large forest patches inferred larger habitat areas of varying microhabitats, abundant habitat resources to support life (e.g., for species that require larger home range to find their specific food), and a refuge from predators (O'Connell, Jackson, & Brooks, 2000). As the size of habitat increases, new species occurrence is encouraged when minimum habitat size requirements are met (Martensen, Pimentel, & Metzger, 2008; Smith, Fahrig, & Francis, 2011). This finding is consistent with (Hill et al., 2011; Şekercioḡlu et al., 2002) wherein both conditions fewer species of birds were found in small sized forest patches. Forest fragmentation that reduces a previously large forest patch into smaller forest pockets may not be adequate in providing resources as compared to a continuous forest habitat. In such cases, avian diversity may be compromised while reducing the number of interior dependent species.

Our result indicated that the density of large trees influenced species richness of forest specialists. Although trees are important for many bird species, native trees have been considered as critical in supporting a large number of forest species (Bolwig et al., 2006). Particularly in this study, we observed that fallow lands and cocoa farms that retained native trees supported more forest specialist as compared with monoculture plantations which had exotic trees. Tree resources are of paramount importance to many wildlife, but particularly so for birds because they provide suitable shelter and nesting locations (Shackleton, Chinyimba, Hebinck, Shackleton, & Kaoma, 2015; Sompud, Mojiol, Gilbert, & Amir, 2014); food (Galetti & Pizo, 2013; Mueller, Lenz, Caprano, Fiedler, & Böhning‐Gaese, 2014) and roost sites (Ssemmanda & Pomeroy, 2014; Villén‐Pérez, Carrascal, & Gordo, 2014). Native trees, in this case, may have been more efficient at providing needed resources for specialist's birds than exotic trees. Our results are confirmed by similarities in the relationship between large trees especially native trees and species assemblages and the benefits above that are derived by birds (Zurita, Rey, Varela, Villagra, & Bellocq, 2006). We emphasize the importance of land use and diversification of native trees for maintaining the populations of forest‐dependent birds in tropical agricultural landscapes such as in our study area and many similar areas in sub‐Saharan Africa (Sekercioglu, 2002; Van Dorp & Opdam, 1987).

Our current study also found a significant number of nectarivorous birds in a monoculture plantation adjacent to a sampled forest patch (Kakum forest reserve). This may be due to higher numbers of flowering trees recorded in the monoculture plantation adjacent the patch as compared to other agricultural land types considered during the sampling period. For nectarivorous birds, the presence of flowering trees implies availability of nectar resources which forms a major component of their diet (Brown, Downs, & Johnson, 2010).

We found more insectivorous birds in the forest interior and adjacent fallow lands compared to forest edges and other land use types. The insectivorous birds observed were mainly ground feeders and ant followers. Fallow lands have been reported to support a high diversity of insect resources (Haaland, Naisbit, & Bersier, 2011). Our finding is consistent with results of earlier studies where the density of ground foliage feeders and insectivorous birds correlated positively with agricultural areas left to fallow (Ford & Bell, 1981). Fallow lands therefore in our study have proved to be profitable and provided feeding substrate for insects. Proper management to provide a continuous flow of resources for insectivorous birds may improve conservation in forest–agricultural landscapes (O'Connor, Shrubb, & Watson, 1990).

Species richness of forest specialists was on the average higher in fallow lands compared to cocoa farms with large trees. This result contradicts studies that suggest the potential of cocoa agroforestry areas to conserve biodiversity than any other anthropogenic land use (Oke & Odebiyi, 2007; Schroth, 2004). Cocoa agroforestry has been credited as more biodiversity friendly compared to other land uses in tropical forest regions (Bos, Steffan‐Dewenter, & Tscharntke, 2007; Van Bael, Bichier, Ochoa, & Greenberg, 2007). Studies conducted in Latin America, Nigeria, and Brazil show that the value of cocoa plantation to support birds (especially many specialists species), insects, and other animals is greater than all other land use types (Rice & Greenberg, 2000; Sambuichi & Haridasan, 2007). On the contrary, many studies have highlighted the conservation value of fallow lands in maintaining biodiversity for insects (Van Emden & Williams, 1974) and birds (Waltert, Mardiastuti, & Mühlenberg, 2004). We argue that fallow lands in this study supported more forest specialists due to their combined structural and compositional similarity to the adjacent forest patches as compared to cocoa farms with large trees. In this study, fallow lands were characterized as >5 years abandoned farm lands. Vegetation characteristics included the presence of large trees which are critical elements of fallow lands in tropical regions (McNeely & Schroth, 2006). Other characteristics include the formation of partial canopies, a rich diversity of shrubs and herbs that formed a multilayered vegetation structure. These often supported a high diversity of insects as such the presence of more insectivorous birds as found in this study. We, therefore, stress that fallow lands may have provided adequate habitat resources to support specialist species and may have accounted for the high richness observed.

Cocoa farms in this study constituted large trees and presence of exotic cocoa plants that formed a two‐layered vertical structure. Shrubs and herbs were almost absent due to the heavy use of agrochemicals in attempts to control weeds and insect pests a common practice in most cocoa farms in Ghana. Agrochemical use often led to increased uniformity and reduction in species and diversity of vegetation (Batáry, Matthiesen, & Tscharntke, 2010). This may have accounted for the reduced species richness of specialist species in comparison with fallow lands.

### 4.3 Implications for conservation

Our results indicate the importance of variations in agricultural land types for biodiversity conservation in tropical forest–agricultural landscapes. Predominantly around many tropical forests are farmlands managed differently depending on the farm type and farmer status. Typical in our study area is an increased use of agrochemicals for weed and pest control and increasing conversion of traditional subsistence farms to commercial cash crops that are usually monocultures. Such agricultural land use practices have drastically changed the formerly biodiversity‐friendly areas that retained native vegetation with occasionally heavy undergrowth. These changes have led to enormous loss of habitat resources for many forest‐dependent species, particularly insect resources for insectivorous birds. This process of matrix modification has impacted bird assemblage structure negatively while rendering habitats unsuitable for native forest specialist species. We suggest that to preserve forest‐dependent birds and other similar biodiversity in forest–agricultural landscapes, proper agricultural land management strategies as well as maintenance of large forest patches should be encouraged. In addition, farmers should be motivated to plant and retain large native trees on their farms to ensure a heterogeneous landscape capable of supporting wildlife.

## CONFLICT OF INTEREST

None declared.

## AUTHOR'S CONTRIBUTION

This study was designed by Justus Precious Deikumah and Richard Kwafo. Logistics and technical support for all aspects of the study were provided by Justus Precious Deikumah. Richard Kwafo collected and analyzed data. Richard Kwafo wrote the manuscript. Justus Precious Deikumah and Vida Asieduwaa Konadu provided critical reviews of the manuscript. All authors approved the final version of the manuscript to be published.
