# Supplementary material for: Land use types influenced avian assemblage structure in a forest–agriculture landscape in Ghana
Source: Ecol Evol. 2017 Sep 18;7(21):8685–97. doi: 10.1002/ece3.3355 (PMC5677502; doi:10.1002/ece3.3355)
Supplement: Supplementary file 1 [file ECE3-7-8685-s001.docx]

**Appendix**

Supplementary data associated with this article can be found at <http://dx.doi.org/10.5061/dryad.v8b0s>.

**Table S1.** List of birds recorded in the study, FHP: forest habitat preference, FP: Foraging preference FHP (FF=forest specialists, F=forest generalists, f=forest visitors, O=open habitat species) FP(C=carnivores, F=frugivores, I=insectivore, P=piscivores, O=omnivores, N=nectarivores, G=granivores)

| **Common name** | **Species** | **Family** | **FHP** | **FP** |
| --- | --- | --- | --- | --- |
| African dwarf kingfisher | *Ceyx lecontei* | Alcedinidae | FF | C |
| African emerald cuckoo | *Chrysococcyx cupreus* | Cuculidae | F | I |
| African green pigeon | *Treron calva* | Columbidae | O | G |
| African goshawk | *Accipiter tachiro* | Accipitridae | F | C |
| African harrier hawk | *Polyboroides typus* | Accipitridae | f | C |
| African paradise flycatcher | *Terpsiphone viridis* | Muscicapidae | F | I |
| African pied hornbill | *Tockus fasciatus* | Bucerotidae | O | O |
| African pygmy kingfisher | *Ceyx pictus* | Alcedinidae | f | I |
| African palm swift | *Cypsiurus parvus* | Apodidae | f | I |
| African reed warbler | *Acrocephalus baeticatus* | Sylviidae | O | G |
| African wood owl | *Strix woodfordi* | Strigidae | FF | C |
| Ahanta francolin | *Francolinus ahantensis* | Phasianidae | O | G |
| Ansorges greenbul | *Andropadus ansorgei* | Pycnonotidae | FF | F |
| Barn swallow | *Hirundo rustica* | Hirundinidae | O | I |
| Bates's swift | *Apus batesi* | Apodidae | F | I |
| Black winged bishop | *Euplectes hordeaceus* | Ploceidae | O | G |
| Black and white flycatcher | *Bias musicus* | Muscicapidae | FF | I |
| Black and white mannikin | *Lonchura cucullata* | Estrildidae | f | G |
| Black bee-eater | *Merops gularis* | Meropidae | FF | I |
| Black coucal | *Centropus grilli* | Cuculidae | O | I |
| Black cuckoo | *Cuculus clamosus* | Cuculidae | F | I |
| Blacked cuckooshrike | *Coracina azurea* | Campephagidae | FF | I |
| Black crowned tchagra | *Tchagra senegala* | Lanidae | f | I |
| Black-rumped waxbill | *Estrlda troglodytes* | Estrildidae | O | G |
| Black dwarf hornbill | *Tockus hartlaubi* | Bucerotidae | FF | I |
| Black capped illadopsis | *Illadopsis cleaveri* | Timalidae | FF | I |
| Black capped apalis | *Apalis nigriceps* | Sylviidae | FF | I |
| Black crowned tchagara | *Tchagra senegala* | Lanidae | f | I |
| Black casqued hornbill | *Ceratogymna atrata* | Bucerotidae | FF | F |
| Black kite | *Milvus migrans* | Accipitridae | f | C |
| Black-winged oriole | *Oriolus nigipennis* | Oriolidae | FF | F |

**Table SI continued**

| **Common name** | **Species** | **Family** | **FHP** | **FP** |
| --- | --- | --- | --- | --- |
| Blue-breasted kingfisher | *Halcyon malimbica* | Alcedinidae | F | C |
| Black-throated coucal | *Centropus leucogaster* | Cuculidae | FF | O |
| Blue-billed malimbe | *Malimbus nitens* | Ploceidae | FF | I |
| Blue-spotted wood dove | *Turtur afer* | Columbidae | f | O |
| Blue-headed wood dove | *Turtur brehmeri* | Columbidae | FF | G |
| Blue-headed coucal | *Centropus monachus* | Cuculidae | F | I |
| Bristle-nosed barbet | *Gymnobucco peli* | Capitonidae | F | F |
| Bronze mannikin | *Lanius cucullata* | Estrildidae | O | G |
| Brown sunbird | *Anthreptes gabonicus* | Nectarinidae | F | N |
| Brown chested alethe | *Alethe poliocephala* | Turdidae | FF | I |
| Brown illadopsis | *Illadopsis fulvescens* | Timalidae | FF | I |
| Blue-throated Brown Sunbird | *Cyanomitra cyanolaema* | Nectarinidae | FF | I |
| Cameroon sombre greenbul | *Andropadus curvirostris* | Pycnonotidae | FF | F |
| Cassin spinetail | *Neafrapus cassini* | Apodidae | f | I |
| Cassin flycatcher | *Muscicapa cassini* | Muscicapidae | FF | I |
| Cattle egret | *Bulbulcus ibis* | Ardeidae | O | P |
| Chestnut wattle eye | *Dyaphorophyia castanea* | Platysteiridae | FF | I |
| Chestnut-breasted negrofinch | *Nigrita bicolor* | Estrildidae | F | F |
| Chestnut-capped flycatcher | *Erythrocercus mccallii* | Muscicapidae | FF | I |
| Chocolate backed kingfisher | *Halcyon badia* | Alcedinidae | FF | I-C |
| Common bulbul | *Pycnonotus barbatus* | Pycnonotidae | f | F |
| Collared sunbird | *Hedydipna collaris* | Nectarinidae | f | F |
| Common fiscal | *Lamprotornis splendidus* | Lanidae | O | I |
| Common wattle eye | *Platysteira cyanea* | Platysteiridae | f | I |
| Copper sunbird | *Platysteira cyanea* | Nectarinidae | FF | N |
| Didric cuckoo | *Chrysococcyx caprius* | Cuculidae | F | I |
| Dusky long-tailed cuckoo | *Cercococcyx mechowi* | Cuculidae | FF | I |
| Double-toothed barbet | *Lybius bidentatus* | Capitonidae | F | F |
| Forest robin | *Stiphrornis erythrothorax* | Turdidae | FF | I |
| Dusky-blue flycatcher | *Muscicapa comitata* | Muscicapidae | F | I |
| Fraser's forest flycatcher | *Fraseria ocreata* | Muscicapidae | FF | I |
| Fire-bellied woodpecker | *Dendropicos pyrrhogaster* | Picidae | FF | I |
| Green sunbird | *Anthreptes rectirostris* | Nectarinidae | FF | N |
| Great blue turaco | *Tauraco persa* | Musophagidae | FF | F |
| Green hylia | *Hylia prasina* | Sylviidae | F | I |
| Grey-backed cameroptera | *Camaroptera brachyura* | Sylviidae | F | I |

**Table SI continued**

| **Common name** | **Species** | **Family** | **FHP** | **FP** |
| --- | --- | --- | --- | --- |
| Grey longbill | *Macrosphenus concolor* | Sylviidae | FF | I |
| Green turaco | *Tauraco persa* | Musophagidae | F | I |
| Green-tailed bristlebill | *Bleda eximia* | Pycnonotidae | FF | O |
| Grey-headed negrofinch | *Nigrita canicapilla* | Estrildidae | f | F |
| Grey-headed bristlebill | *Bleda canicapilla* | Pycnonotidae | F | O |
| Hairy-breasted barbet | *Tricholaema hirsuta* | Capitonidae | FF | F |
| Hooded vulture | *Necrosyrtes monachus* | Accipitridae | O | O |
| Honey guide greenbul | *Baeopogon indicator* | Pycnonotidae | FF | F |
| Icterine greenbul | *Phyllastrephus icterinus* | Pycnonotidae | FF | I |
| Kemps longbill | *Macrosphenus kempi* | Sylviidae | FF | I |
| Klass's cuckoo | *Chrysococcyx klaas* | Cuculidae | F | I |
| Johanna's sunbird | *Cinnyris johannae* | Nectarinidae | FF | F |
| Little greenbul | *Andropadus virens* | Pycnonotidae | F | F |
| Levaillant's cuckoo | *Oxylophus levaillantii* | Cuculidae | f | I |
| Little green sunbird | *Anthreptes seimundi* | Nectarinidae | F | N |
| Little bee-eater | *Merops pusillus* | Meropidae | f | I |
| Little grey greenbul | *Andropadus gracilis* | Pycnonotidae | FF | F |
| Little swift | *Apus affinis* | Apodidae | f | I |
| Naked-faced barbet | *Gymnobucco calvus* | Capitonidae | F | F |
| Northern grey-headed sparrow | *Passer griseus* | Passeridae | O | G |
| Olive-bellied sunbird | *Cinnyris chloropygius* | Nectarinidae | F | N |
| Olive sunbird | *Cyanomitra olivacea* | Nectarinidae | F | N |
| Olive-green camaroptera | *Camaroptera chloronota* | Sylviidae | F | I |
| Olive long-tailed cuckoo | *Cercococcyx olivinus* | Cuculidae | FF | C |
| Orange cheeked waxbil | *Estrilda melpoda* | Estrildidae | O | G |
| Oriole warbler | *Hypergerus atriceps* | Sylviidae | F | I |
| Pale-breasted illadopsis | *Illadopsis rufescens* | Timalidae | FF | I |
| Pallid harrier | *Circus macrourus* | Accipitridae | O | C |
| Palm swift | *Cypsiurus parvus* | Apodidae | f | I |
| Pied crow | *Corvus albus* | Corvidae | O | O |
| Pin-tailed whydah | *Vidua macroura* | Viduidae | f | F |
| Piping hornbill | *Bycanistes fistulator* | Bucerotidae | FF | F |
| Purple-throated cuckooshrike | *Campephaga quiscalina* | Campephagidae | F | I |
| Red-bellied paradise flycatcher | *Terpsiphone rufiventer* | Muscicapidae | F | I |
| Red-billed dwarf hornbill | *Tockus camurus* | Bucerotidae | F | I |
| Red-checked wattle eye | *Dyaphorophyia blissetti* | Platysteiridae | FF | I |

**Table SI continued**

| **Common name** | **Species** | **Family** | **FHP** | **FP** |
| --- | --- | --- | --- | --- |
| Red-chested cuckoo | *Cuculus solitarius* | Cuculidae | FF | I |
| Red-eyed dove | *Streptopelia semitorquata* | Columbidae | f | F |
| Red-faced cisticola | *Cisticola erythrops* | Cisticolidae | f | G |
| Red-rumped tinkerbird | *Pogoniulus atroflavus* | Capitonidae | FF | F |
| Red-tailed bristlebill | *Bleda syndactyla* | Pycnonotidae | FF | O |
| Rufous-sided broadbill | *Smithornis rufolateralis* | Eurylaimidae | FF | I |
| Rufous-winged illadopsis | *Illadopsis puveli* | Timalidae | FF | F |
| Rosy bee-eater | *Merops malimbicus* | Meropidae | FF | I |
| Sabine spinetail | *Rhaphidura sabini* | Apodidae | f | I |
| Sabine puffback | *Dryoscopus sabini* | Malaconotidae | F | I |
| Scarlet-chested sunbird | *Chalcomitra sengalensis* | Nectarinidae | f | N |
| Senegal coucal | *Centropus senegalensis* | Cuculidae | f | I |
| Simple leavelove | *Chlorocichla simplex* | Pycnonotidae | F | I |
| Sharpe's apalis | *Apalis sharpii* | Cisticolidae | FF | I |
| Shining drongo | *Dicrurus atripennis* | Dicruridae | FF | I |
| Speckled tinker bird | *Pogoniulus scolopaceus* | Capitonidae | F | I |
| Splendid sunbird | *Cinnyris coccinigatrus* | Nectarinidae | O | N |
| Spotted greenbul | *Ixonotus guttatus* | Pycnonotidae | FF | I |
| Splendid glossy starling | *Lamprotornis cupreocauda* | Sturnidae | O | F |
| Superb sunbird | *Cinnyris superbus* | Nectarinidae | f | N |
| Swamp palm bulbul | *Thescelocichla leucopleura* | Pycnonotidae | O | F |
| Tambourine dove | *Turtur tympanistria* | Columbidae | f | G |
| Tawny-flanked prinia | *Prinia subflava* | Sylviidae | O | I |
| Tit hylia | *Pholidornis rushiae* | Remizidae | FF | F |
| Vieillot’s black weaver | *Ploceus nigerrimus* | Ploceidae | f | I |
| Velvet-mantled drongo | *Dicrurus modestus* | Dicruridae | F | I |
| Tropical boubou | *Laniarius aethiopicus* | Malaconotidae | f | I |
| Village weaver | *Ploceus cucullatus* | Ploceidae | O | I |
| Violet turaco | *Musophaga violacea* | Musophagidae | F | I |
| Western bearded greenbul | *Criniger barbatus* | Pycnonotidae | FF | F |
| Western black-headed oriole | *Oriolus brachyrhynchus* | Oriolidae | F | F |
| Western bluebill | *Spermophaga haematina* | Ploceidae | F | F |
| Western bronze-naped pigeon | *Columba iriditorques* | Columbidae | FF | F |
| Western grey plantain-eater | *Criniger piscator* | Musophagidae | O | F |
| Western nicator | *Nicator chloris* | Pycnonotidae | F | I |
| Whistling cisticola | *Cisticola lateralis* | Cisticolidae | f | G |

**Table SI continued**

| **Common name** | **Species** | **Family** | **FHP** | **FP** |
| --- | --- | --- | --- | --- |
| White-tailed alethe | *Alethe diademata* | Turdidae | FF | I |
| White-crested hornbill | *Tropicranus albocristatus* | Bucerotidae | FF | O |
| White-tailed ant thrush | *Neocossyphus poensis* | Turdidae | FF | I |
| White-throated bee-eater | *Merops albicollis* | Meropidae | f | I |
| Yellowbill | *Ceuthmochares aereus* | Cuculidae | F | I |
| Woodland kingfisher | *Halcyon senegalensis* | Alcedinidae | f | C |
| Yellow-billed barbet | *Trachylaemus purpuratus* | Capitonidae | f | F |
| Yellow-billed turaco | *Tauraco macrorhynchus* | Musophagidae | F | F |
| Yellow-browed camaroptera | *Camaroptera superciliaris* | Sylviidae | F | I |
| Yellow-fronted tinkerbird | *Pogoniulus chrysoconus* | Capitonidae | F | F |
| Yellow-rumped tinkerbird | *Pogoniulus atroflavus* | Capitonidae | FF | F |
| Yellow-spotted barbet | *Buccanodon duchaillui* | Capitonidae | FF | F |
| Yellow spotted tinker | *Pogoniulus scolopaceus* | Capitonidae | F | F |
| Yellow-whiskered greenbul | *Andropadus latirostris* | Pycnonotidae | F | O |
| Yellow-throated tinkerbird | *Pogoniulus subsulphureus* | Capitonidae | FF | F |

**S2 Table.** Correlation matrix of explanatory variables. Coefficients in **bold** shows highly correlated variables.

| **Explanatory variables** | 1 | 2 | 3 | 4 | 5 | 6 | 7 |
| --- | --- | --- | --- | --- | --- | --- | --- |
| 1. Large trees | 1 |  |  |  |  |  |  |
| 2. Fruiting trees | 0.05 | 1 |  |  |  |  |  |
| 3. Flowering trees | -0.07 | **0.89** | 1 |  |  |  |  |
| 4. Canopy cover | **0.78** | 0.10 | -0.04 | 1 |  |  |  |
| 5. Shrub density | **0.65** | -0.11 | -0.17 | **0.60** | 1 |  |  |
| 6. Ground cover | 0.19 | -0.18 | -0.17 | -0.04 | 0.18 | 1 |  |
| 7. Patch size | -0.01 | -0.01 | -0.04 | 0.14 | -0.05 | -0.07 | 1 |


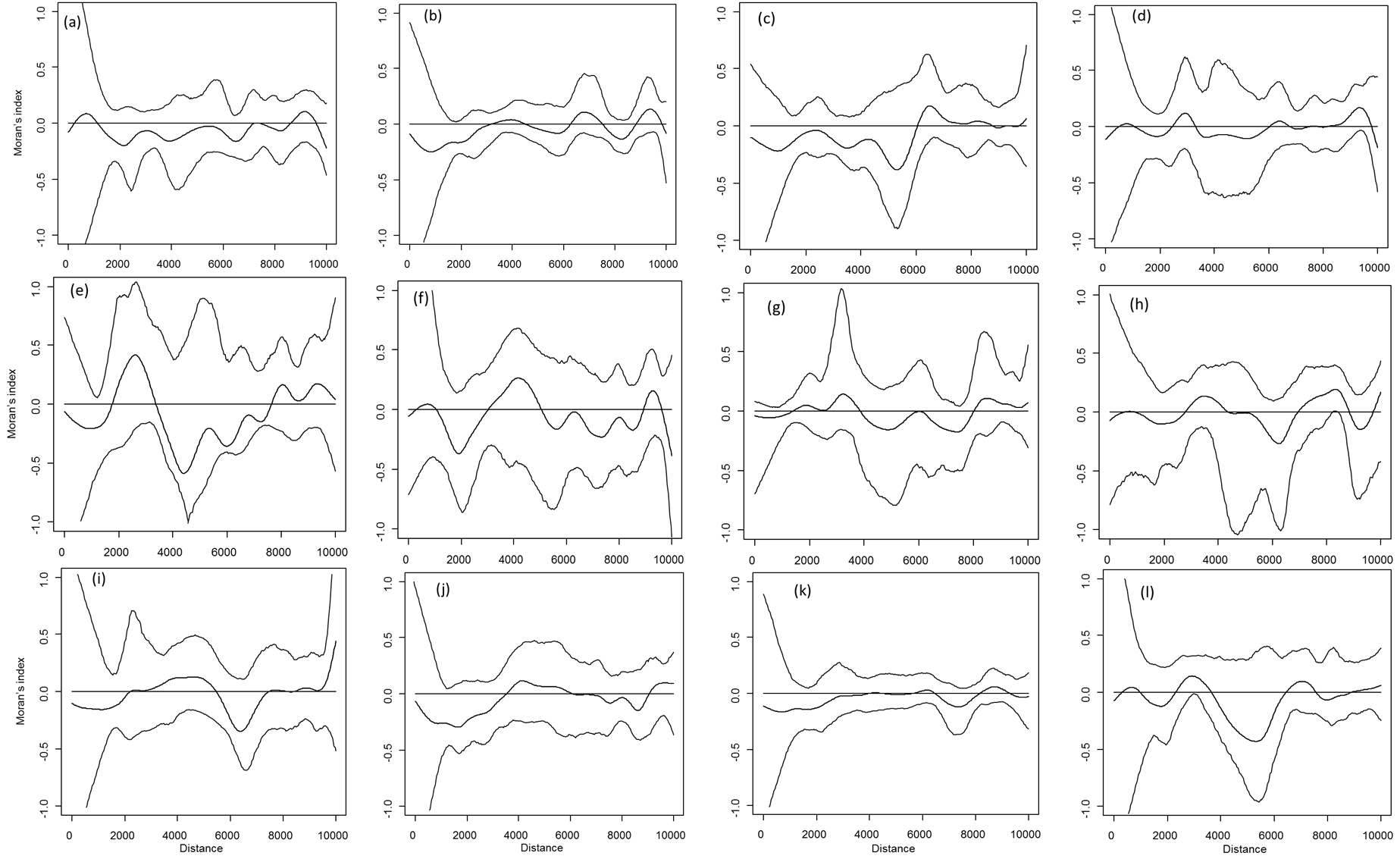


**S1 Fig.** Spline correlogram of final model residuals for responses showing Moran’s similarity indices at different distance from study sites; (a) observed species richness (b) estimated species richness (chao1) (c) Simpson’s diversity index (d) species evenness (e) forest specialists (f) forest generalists (g) forest visitors (h) open habitat species (i) insectivores (j) frugivores (k) granivores (l) nectarivores
